# Rab GTPases Regulate Endothelial Cell Protein C Receptor-Mediated Endocytosis and Trafficking of Factor VIIa

**DOI:** 10.1371/journal.pone.0059304

**Published:** 2013-03-15

**Authors:** Ramesh C. Nayak, Shiva Keshava, Charles T. Esmon, Usha R. Pendurthi, L. Vijaya Mohan Rao

**Affiliations:** 1 Department of Cellular and Molecular Biology, The University of Texas Health Science Center at Tyler, Tyler, Texas, United States of America; 2 Coagulation Biology Laboratory, Oklahoma Medical Research Foundation, Howard Hughes Medical Institute, Oklahoma City, Oklahoma, United States of America; University of Illinois College of Medicine, United States of America

## Abstract

Recent studies have established that factor VIIa (FVIIa) binds to the endothelial cell protein C receptor (EPCR). FVIIa binding to EPCR may promote the endocytosis of this receptor/ligand complex. Rab GTPases are known to play a crucial role in the endocytic and exocytic pathways of receptors or receptor/ligand complexes. The present study was undertaken to investigate the role of Rab GTPases in the intracellular trafficking of EPCR and FVIIa. CHO-EPCR cells and human umbilical vein endothelial cells (HUVEC) were transduced with recombinant adenoviral vectors to express wild-type, constitutively active, or dominant negative mutant of various Rab GTPases. Cells were exposed to FVIIa conjugated with AF488 fluorescent probe (AF488-FVIIa), and intracellular trafficking of FVIIa, EPCR, and Rab proteins was evaluated by immunofluorescence confocal microscopy. In cells expressing wild-type or constitutively active Rab4A, internalized AF488-FVIIa accumulated in early/sorting endosomes and its entry into the recycling endosomal compartment (REC) was inhibited. Expression of constitutively active Rab5A induced large endosomal structures beneath the plasma membrane where EPCR and FVIIa accumulated. Dominant negative Rab5A inhibited the endocytosis of EPCR-FVIIa. Expression of constitutively active Rab11 resulted in retention of accumulated AF488-FVIIa in the REC, whereas expression of a dominant negative form of Rab11 led to accumulation of internalized FVIIa in the cytoplasm and prevented entry of internalized FVIIa into the REC. Expression of dominant negative Rab11 also inhibited the transport of FVIIa across the endothelium. Overall our data show that Rab GTPases regulate the internalization and intracellular trafficking of EPCR-FVIIa.

## Introduction

The endothelial cell protein C receptor (EPCR) is the cellular receptor for protein C (PC) and activated protein C (APC), and is mainly present on the endothelial cell lining of larger blood vessels [1], [2]. EPCR is primarily localized on the cell surface in membrane microdomains that are positive for caveolin-1, but a small fraction of EPCR is also localized intracellularly, particularly in the pericentriolar recycling endosomal compartment (REC) at the juxtanuclear region [3]. Recently, we and others have shown that EPCR also functions as a cellular receptor for coagulation factor VII (FVII) and activated factor VII (FVIIa) [4]–[6]. Our studies also revealed that FVIIa or APC binding to EPCR promotes the internalization of EPCR. EPCR and the bound ligands are endocytosed rapidly via dynamin- and caveolae-dependent pathways [3]. The endocytosed receptor-ligand complexes accumulate in the recycling compartment before being targeted back to the cell surface. EPCR-mediated endocytosis is thought to facilitate the transcytosis of FVIIa [3]. At present, the endocytic signaling pathways that mediate internalization of EPCR and intracellular trafficking of the endocytosed EPCR-FVIIa complex are unknown.

A subfamily of Ras-like small GTPases, termed as Rab GTPases, have been shown to play a critical regulatory role in both endocytic and exocytic pathways of protein trafficking by regulating vesicular membrane transport and membrane fusion events [7]–[9]. Although some overlap exists, different Rab GTPases localize to different distinct endosomal compartments and act as key regulators of the vesicular trafficking between these compartments [8]–[10]. Rab5 is localized to the plasma membrane, clathrin-coated vesicles, and early endosomes [11]. Rab 5 is shown to regulate both the constitutive and ligand-induced internalization of cell surface receptors from the plasma membrane to the early endosomal compartment, and facilitates the homotypic fusion of early endosomes [12], [13]. Rab4 exhibits overlapping distribution with Rab5 in early and recycling endosomes, and controls the rapid recycling of cargo proteins directly back to the cell surface from Rab4/Rab5 positive endosomal structures [14]. Rab4 also regulates the slow recycling of cargo via Rab11 positive recycling endosomes [15]–[17]. Rab11 is generally localized to perinuclear recycling endosomes and considered to control slow endosomal recycling from the recycling endosomal compartment to the cell surface [18]–[20]. Rab11 may also regulate the transcytotic migration of internalized ligands from apical to basal surfaces in polarized epithelial cells [21]. Rab7 is localized to late endosomes and to the lysosomal compartment, and thus this Rab GTPase is thought to regulate vesicular traffic between late endosomes and lysosomes [22], [23]. Although the role of Rab4, Rab5, Rab7, and Rab11 in regulating endocytosis as well as intracellular trafficking has been studied extensively with respect to transferrin receptor and few G-protein coupled receptors [see rev [9], [15], [19]], the role of these Rab GTPases in regulating endocytosis and intracellular trafficking of EPCR has not been examined.

In the present study, we investigated whether Rab GTPases regulate the internalization, intracellular trafficking, and recycling of EPCR and EPCR bound ligand. We show that Rab 4, Rab 5, and Rab 11 control the intracellular trafficking of EPCR and FVIIa at different stages. Overall, our data suggest that Rab GTPases play important roles in the endosomal sorting/recycling of EPCR and provide information on a potential mechanism for regulation of EPCR levels on the cell surface and EPCR-dependent transcytosis.

## Materials and Methods

### Reagents

Rabbit polyclonal antibodies against Rab5, Rab4, Rab7 and Rab11 were purchased from Santa Cruz Biotechnology Inc. (Santa Cruz, CA). Secondary antibodies conjugated with Oregon Green or Rhodamine Red, and Alexa Fluor 488 (AF488) labeling kit were obtained from Invitrogen Corp. (Carlsbad, CA). Mouse monoclonal antibodies against human EPCR (JRK-1494/blocking mAb and JRK-1500/non-blocking mAb) were prepared as described earlier [24]. Recombinant human FVIIa was from Novo Nordisk A/S (Malov, Denmark) and recombinant activated protein C (Xigris) was from Eli Lilly (Indianapolis, IN).

### Cell Culture

Primary human umbilical vein endothelial cells (HUVEC), EBM-2 basal medium, and growth supplements were purchased from Lonza (Walkersville, MD). Endothelial cells were cultured in EBM-2 basal medium supplemented with growth supplements, 1% penicillin/streptomycin, and 5% fetal bovine serum. Generation of CHO cells stably expressing EPCR (CHO-EPCR) was described previously [5]. Both wild-type CHO and CHO-EPCR cells were cultured in Ham’s F12 medium containing 1% penicillin/streptomycin and supplemented with 10% fetal bovine serum. HEK 293 cells were cultured in DMEM medium containing 1% penicillin/streptomycin and supplemented with 10% fetal bovine serum.

### Plasmid Constructs

Wild type (WT) Rab5A, its constitutively active (CA) form (Rab5A^Q67L^), and dominant negative (DN) variant (Rab5A^S34N^) were kindly provided by Brian Knoll (University of Houston, Houston, TX). Rab11 and its variants, Rab11^Q70L^ and Rab11^S25N^, were provided by David Sabatini (New York University School of Medicine, New York, NY). Rab4A cDNA was provided by Marino Zerial (Max Planck Institute of Molecular Cell Biology and Genetics, Dresden, Germany). Rab7 and its variants, Rab7^Q67L^ and Rab7^T22N^, were provided by Juan Bonifacino (National Institute of Child Health & Human Development, Bethesda, MA). All of the above plasmid inserts were transferred into adenoviral shuttle vector pacAD5CMV K-N pA using standard cloning techniques. Rab4A^Q67L^ and Rab4A^S34N^ variants were generated by site-directed mutagenesis method using “Quick change II XL” site directed mutagenesis kit (Stratagene, La Jolla, CA) and pacAD5CMV K-N- pA Rab4A as the template.

### Generation of High-titer Adenoviruses of Rab4A, Rab5A, Rab7, and Rab11 Variants

HEK 293 cells seeded in 60 mm dishes (80% confluent) were cotransfected with 1 µg of adenoviral backbone DNA and 5 µg of pac1-digested linearized pacAD5CMV K-N pA containing Rab DNA using Fugene HD transfection reagent according to the manufacturer’s protocol (Roche Diagnostics Corp. Indianapolis, IN). After 7–8 days of transfection, HEK 293 cells showing cytopathic effects were lysed by repeated freeze/thaw cycles and centrifuged at 3,000×g to collect the supernatant containing primary adenoviral stock, and this primary adenoviral stock was used to infect HEK 293 cells in 6–8 T-75 flasks to generate high titer viruses. Viral titers were determined according to the manufacturer’s protocol using “Quick Titer Adenovirus Titer Immunoassay” kit (Cell Biolabs, Inc. San Diego, CA). In general, HUVEC were transduced with 20 MOI/cell whereas 50 MOI/cell were used for transduction in CHO-EPCR cells.

### Labeling of FVIIa with AF488 Fluorescent Probe

FVIIa was labeled with AF488 fluorescent probe using micro scale protein labeling kit (Invitrogen Corporation, Carlsbad, CA) as described recently [25]. Approximately 100 µg of protein was used for each labeling, and the degree of labeling (moles dye/mole protein) was very similar (3.7–4.0) among different batches of labeling.

### Immunofluorescence-based Internalization Assay

CHO-EPCR cells cultured on fibronectin-coated glass cover slips were infected for 48 h with adenoviruses encoding one of the Rab variants or control adenovirus. After 48 h, the cells were washed with buffer B (10 mM HEPES, 0.15 M NaCl, 4 mM KCl, 11 mM glucose, pH 7.5 buffer containing 5 mM CaCl_2_, 1.0 mM MgCl_2_, and 1 mg/ml BSA), and incubated with AF488-FVIIa (50 nM) in buffer B at 4°C (on an ice-bath in a cold room) for 1 h to allow binding of the ligand to EPCR with no or minimal internalization of the bound ligand. At the end of the 1 h incubation, the unbound ligand was removed; cells were washed twice with cold buffer B, and then were transferred to 37°C to induce internalization. At varying time intervals the cells were fixed, permeabilized, and processed for immunofluorescence confocal microscopy. For steady-state internalization studies, HUVEC were incubated with AF488-FVIIa (50 nM) at 37°C for a fixed time.

### Immunofluorescence Confocal Microscopy

Fixed and permeabilized cells were subjected to immunostaining for EPCR and various Rabs as described recently by us [3]. Briefly, cells were fixed with 4% paraformaldehyde in PBS for 1 h at 4°C, permeabilized with 0.05% Triton X-100 in PBS for 10 min, and blocked with 3% goat serum for 1 h at room temperature. The permeabilized cells were incubated with EPCR mAb and/or rabbit polyclonal antibody specific to one of the Rabs overnight at 4°C. After removing the unbound primary antibodies, the cells were washed twice with PBS, and incubated with Rhodamine Red-conjugated (excitation/emission wavelength, 590/620 nm), Oregon Green-conjugated (excitation/emission wavelength 490/510 nm), or AF647-conjugated (excitation/emission wavelength, 650/668 nm) anti-rabbit or anti-mouse IgG for 60 min at room temperature. In some experiments, nuclei were also stained with 4,6 diamidion 2-phenylindole (DAPI, excitation/emission wavelength 370/450 nm). The cells were washed and the cover slips were mounted on a glass slide using aqueous gel mounting media (FLUROGEL, Electron Microscopy Services, Hatfield, MA) containing anti-fading agent. The immunostained cells were imaged and analyzed using Zeiss confocal system (LSM 510 META) equipped with an inverted microscope (Axio Observer Z1) [3]. Based on our earlier study [3], EPCR-FVIIa localized at the juxtanuclear region inside the cell was considered as EPCR-FVIIa in the recycling compartment (REC). To quantify, FVIIa levels in the REC, a region of interest (ROI) was made encircling the juxtanuclear region, and the mean fluorescence intensity of AF488-FVIIa within the ROI was determined by using ZEN 2009 software (Carl Zeiss). To measure recycling of FVIIa to the plasma membrane, several ROI were created randomly by outlining the plasma membrane and the mean fluorescence intensity of AF488-FVIIa in these regions was measured as above.

### Radioactivity-based Internalization, Degradation and Recycling Assays

Internalization, recycling, and degradation of ^125^I-labeled FVIIa were determined as described recently [5]. EPCR levels at the cell surface were determined by incubating the cells with ^125^I-labeled EPCR mAb for 2 h at 4°C.

### Transcytosis

A transwell permeable system (3-µm pore size, polyester membrane, 12-mm diameter; Corning, NY) was used to evaluate the transport of FVIIa from apical to basal surface. Briefly, upper chamber inserts were coated with 0.05% fibronectin (Sigma, St Louis, MO USA) for 30 min, washed once with serum-free medium, and air-dried. HUVEC were seeded in the upper chamber (50,000 cells/well) and allowed to grow for 48 h in EBM-2 growth medium. After 48 h, HUVEC were infected with either control adenovirus or adenovirus encoding wild-type, constitutively active or dominant negative Rab11 (20 moi/cell). After culturing cells further for 72 h, the cells were washed twice with serum-free medium, and serum-free EBM-2 medium supplemented with 2% BSA was added to both upper and bottom chambers. FVIIa (10 nM) was added to the upper chamber. The cells were allowed to incubate for 2 h at 37°C and 5% CO_2_. At the end of 2 h, the medium from the bottom chamber was removed and the FVIIa that transcytosed into the bottom chamber was determined in FXa generation assay using saturating concentrations of relipidated TF.

### Data Collection and Statistical Analysis

The images were processed using LSM Zen 2009 (Zeiss) software and imported to Adobe Photoshop for compilation of figures. When mean fluorescence was determined, typically fluorescence values of 10 to 20 ROI were used for determining FVIIa accumulation at the REC, and 30 to 50 ROI for determining FVIIa levels at the cell surface (for recycling to the plasma membrane). Unpaired t-test was used to calculate whether an experimental value significantly differs from the control value.

## Results

### FVIIa Internalized via EPCR-mediated Endocytosis Specifically Colocalizes with Rab4A, Rab5A, and Rab11 at Different Times

**Figure 1:**
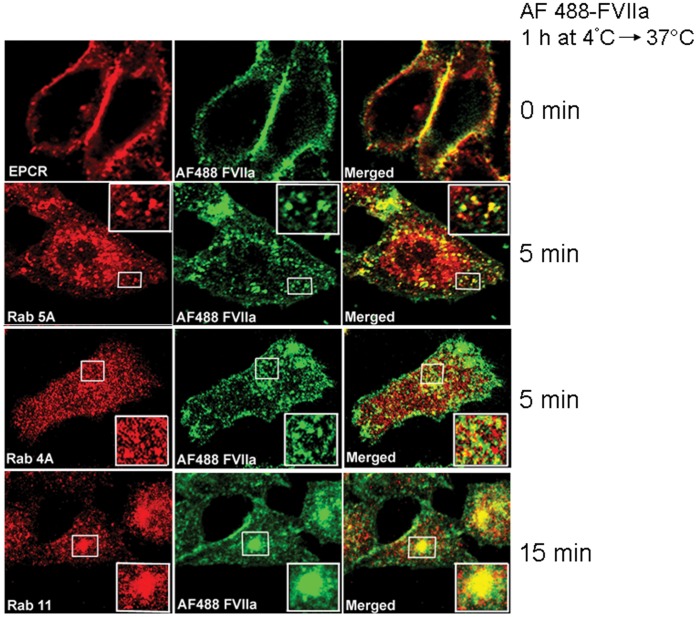
Colocalization of internalized FVIIa and Rab GTPases. CHO-EPCR cells were exposed to AF488-FVIIa (50 nM) for 1 h at 4°C. At the end of 1 h, the supernatant was removed, cells were washed quickly with Ca^2+^/Mg^2+^-containing buffer to remove the unbound ligand and then transferred to 37°C to induce internalization of the surface bound ligand. At the end of 0, 5, and 15 min, cells were fixed, permeabilized, and immunostained with mouse monoclonal or rabbit polyclonal antibodies against EPCR, Rab5A, Rab4A, or Rab11 followed by Rhodamine Red-labeled anti-mouse/anti-rabbit IgG as a secondary reporter antibody. The cells were imaged as described in methods. Left panel, immunostaining of EPCR, Rab5A, Rab4A or Rab11; middle panel, fluorescence of AF488-FVIIa. The right panel depicts the merged images of left and middle panels. The insets show the magnified view of the boxed regions.

Our recent studies [3] demonstrate that EPCR occupancy by its ligands, FVIIa or APC, results in the internalization of the receptor-ligand complex. Following endocytosis, the complex first enters into an early endosomal compartment and then reaches the Rab11 positive recycling endosomal compartment (REC), before being mostly sorted back to the surface. The FVIIa binding and internalization noted in HUVEC and CHO-EPCR cells by confocal microscopy was entirely EPCR-specific as no visible FVIIa binding and internalization was observed if the cells were pretreated with EPCR blocking mAb [3], [26]. Thus, these cell model systems are suitable for investigating the role(s) of various Rab GTPases on EPCR-dependent endocytosis and intracellular trafficking of FVIIa. To investigate whether Rab GTPases regulate the internalization and intracellular trafficking of endocytosed EPCR-FVIIa complexes, we initially analyzed the localization of internalized AF488-FVIIa with various Rab GTPases of the endocytic pathway in CHO-EPCR cells and HUVEC by immunofluorescence confocal microscopy. AF488-FVIIa was first allowed to bind CHO-EPCR cells at 4°C before promoting internalization at 37°C. As expected, AF488-FVIIa associated with cells at 4°C is fully colocalized with EPCR on the cell surface. Following the inducement of internalization by raising the temperature to 37°C, the internalized AF488-FVIIa colocalizes with Rab5A and Rab4A positive endosomal compartments beneath the plasma membrane within 5 min, indicating effective entry of the internalized ligand into the early/sorting endosomes. However, the extent of colocalization of AF488-FVIIa with Rab4A positive endosomes was much lower than that which was observed with Rab5A, indicating that only a fraction of the internalized FVIIa goes directly to sorting endosomes from early endosomes. Consistent with our earlier finding [3], most of the internalized AF488-FVIIa reaches the perinuclear REC within 15 min, where it colocalizes with Rab11 (Fig. 1). We found negligible colocalization of AF488-FVIIa with either the late endosomal marker Rab7 or the lysosomal marker LAMP1 at any time period of the experiment (0 to 60 min; data not shown). A similar localization pattern of internalized AF488-FVIIa with Rab GTPases was also found in HUVEC (data not shown).

### Rab5A Regulates the Internalization of AF488-FVIIa from the Plasma Membrane, and its Entry into Early Endosomes

**Figure 2:**
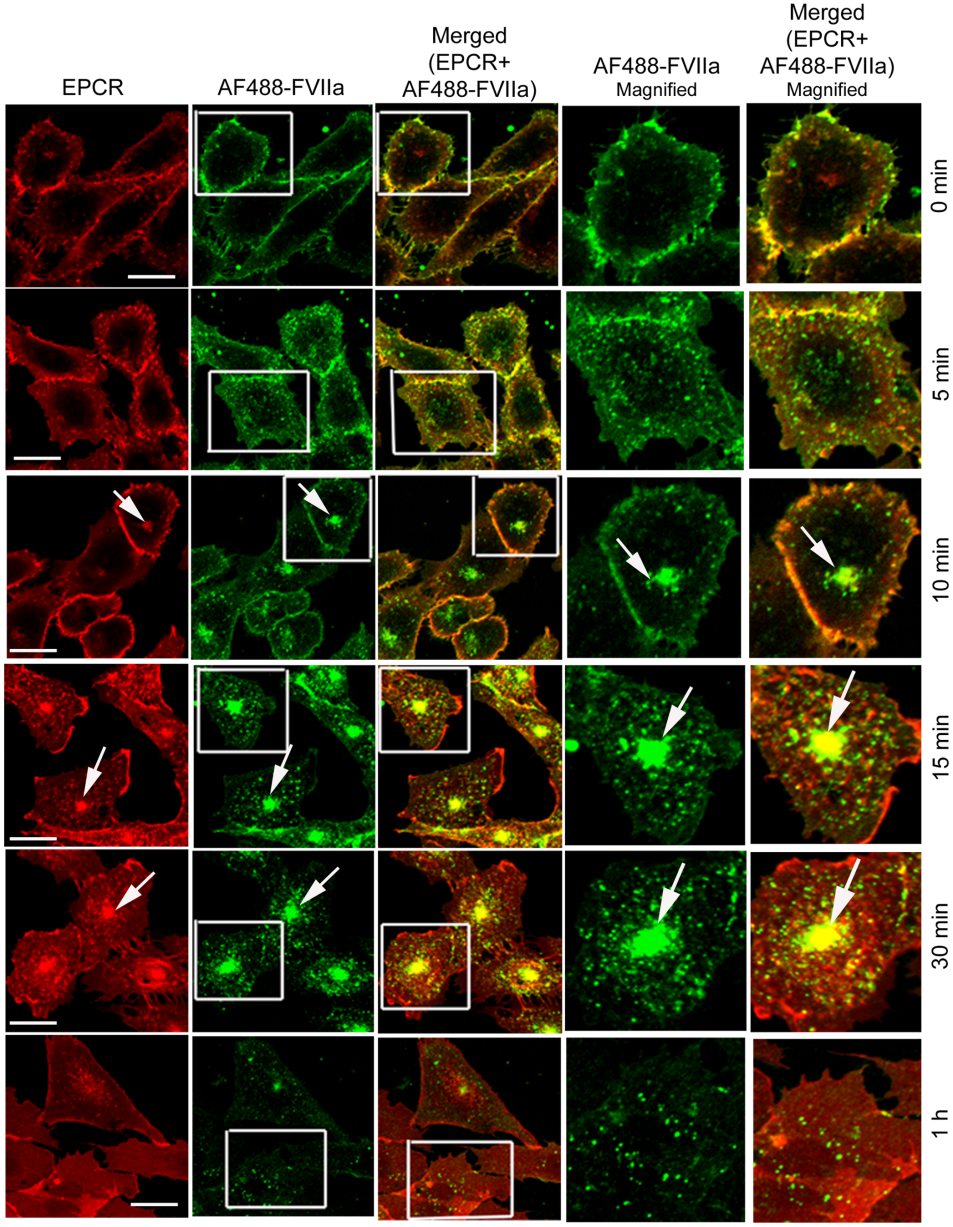
Internalization and trafficking of EPCR and FVIIa in EPCR expressing cells. CHO-EPCR cells transduced with control adenoviruses were incubated with AF488-FVIIa (50 nM) for 1 h at 4°C. After removing the unbound ligand, cells were transferred to 37°C to induce internalization of the surface bound ligand. The cells were immunostained for EPCR and analyzed for immunofluorescence of EPCR and fluorescence of AF488-FVIIa. The two right panels are digitally enlarged images of the inset, and the arrow indicates the accumulation of AF488-FVIIa and EPCR in the REC. Please note that in this and other figures involving CHO-EPCR cells, the images in top three panels were a single chosen section from z-stack and the images in the bottom three panels were reconstructed composite of all z-stacks. We chose this presentation to show FVIIa trafficking from the surface to REC via endosomes more illustratively, as different compartments reside in different planes. Bar scale shown here and in other figures for CHO-EPCR cells represent 10 µm.

To investigate the role of various Rab GTPases in EPCR-mediated endocytosis and intracellular trafficking of FVIIa, CHO-EPCR cells were transduced with either control adenoviruses or adenoviruses encoding WT, CA, or DN variants of Rab4A, Rab5A, Rab7, or Rab11. The transduced cells were exposed to AF488-FVIIa, and the endocytosis and intracellular trafficking of EPCR and FVIIa were analyzed. In cells transfected with control adenovirus, at 4°C, AF488-FVIIa bound to the cell surface and colocalized exclusively with EPCR at the cell surface (Fig. 2). The surface bound ligand was internalized only after the cells were warmed to 37°C. Internalized FVIIa was organized in endosomal structures beneath the plasma membrane within 5 to 10 min following the onset of internalization. After a 15 min time period, the internalized FVIIa along with EPCR accumulated in the REC. By 1 h, AF488-FVIIa that had been accumulated in the REC disappeared, suggesting that FVIIa accumulated in the REC was recycled back to the cell surface or routed to other intracellular compartments (Fig. 2).

**Figure 3:**
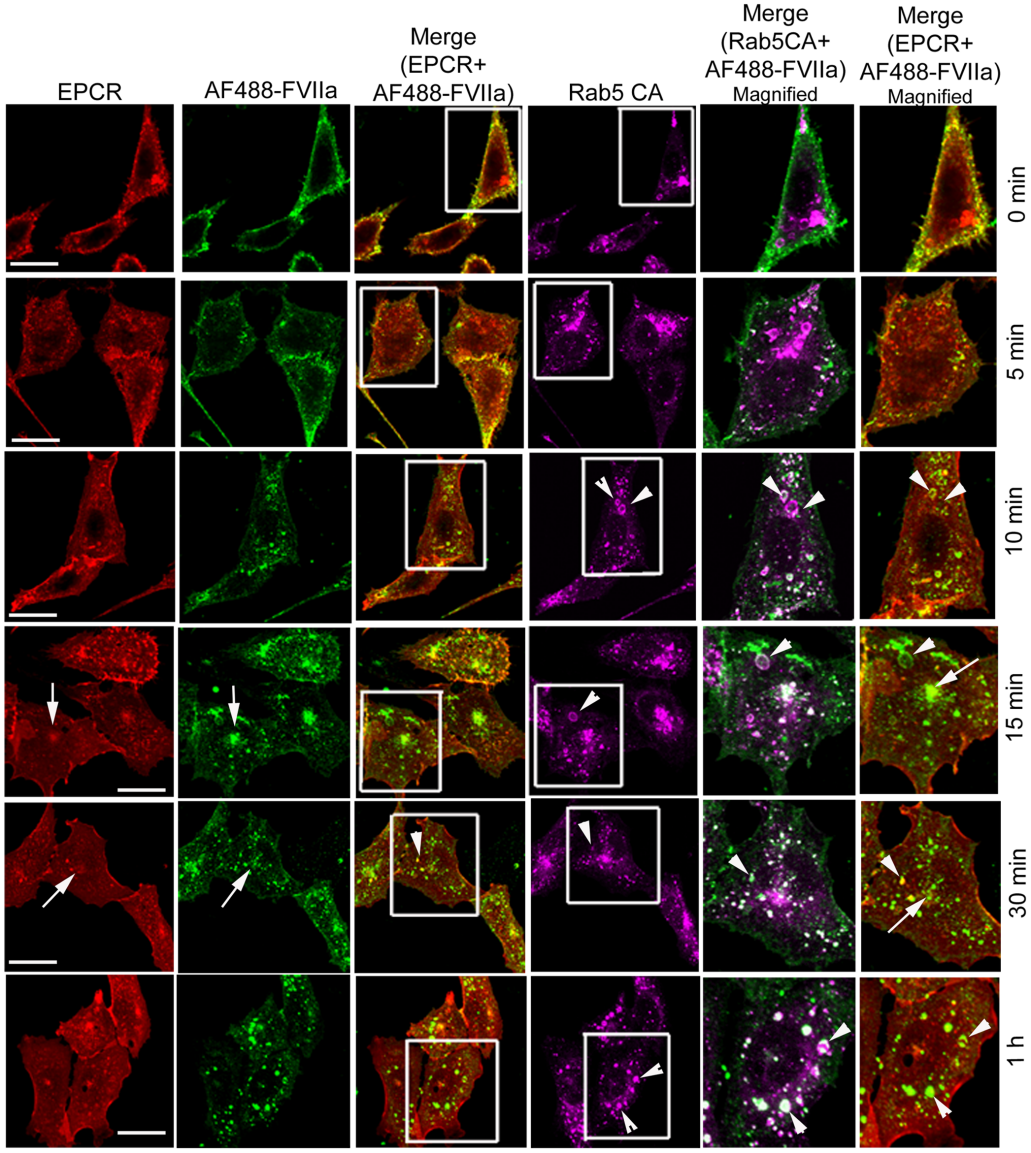
Rab5A regulates the internalization and trafficking of FVIIa through early endosomes. CHO-EPCR cells transduced with recombinant adenoviruses to express constitutively active Rab5A were incubated with AF488-FVIIa (50 nM) for 1 h at 4°C. After removing the unbound ligand, cells were transferred to 37°C to induce internalization of the surface bound ligand. The cells were immunostained for EPCR and Rab5, and analyzed for immunofluorescence of EPCR and Rab. The cells were also analyzed for fluorescence of AF488-FVIIa. The two right panels are the digitally enlarged images of a small portion of the merged image of AF488-FVIIa with Rab5 or EPCR staining, respectively. The arrow indicates the accumulation of AF488-FVIIa in the REC and the arrow head shows the trapping of AF488-FVIIa in the enlarged endosomal structures.

**Figure 4:**
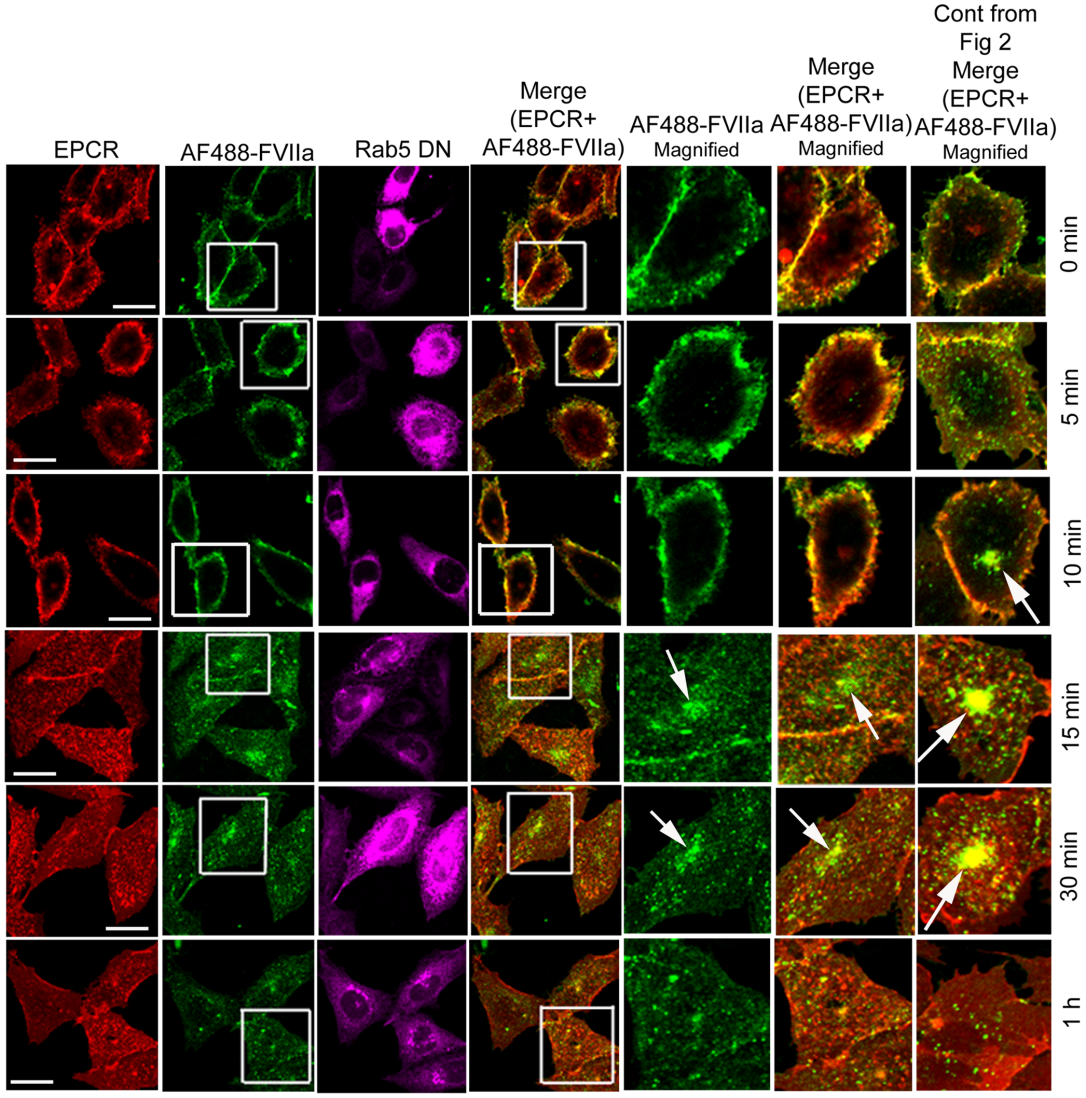
Overexpression of dominant negative Rab5A variant inhibits the endocytosis of FVIIa-EPCR in CHO-EPCR cells. CHO-EPCR cells transduced to express dominant negative Rab5A mutant were incubated with AF488-FVIIa (50 nM) for 1 h at 4°C, and internalization was induced at 37°C as described in Fig. 3. The cells were immunostained for EPCR and Rab5. They were then analyzed for immunofluorescence of EPCR and Rab, and fluorescence of AF488-FVIIa. The 5^th^ and 6^th^ panels are the digitally enlarged images of the insets of the 2^nd^ and 4^th^ panels, respectively. The extreme right panel was the same panel that was shown in Fig. 3, but re-inserted here to illustrate differences in AF488-FVIIa and EPCR accumulation in the REC between control and Rab5A DN expressing cells. Therrow indicates the accumulation of AF488-FVIIa at REC.

**Figure 5:**
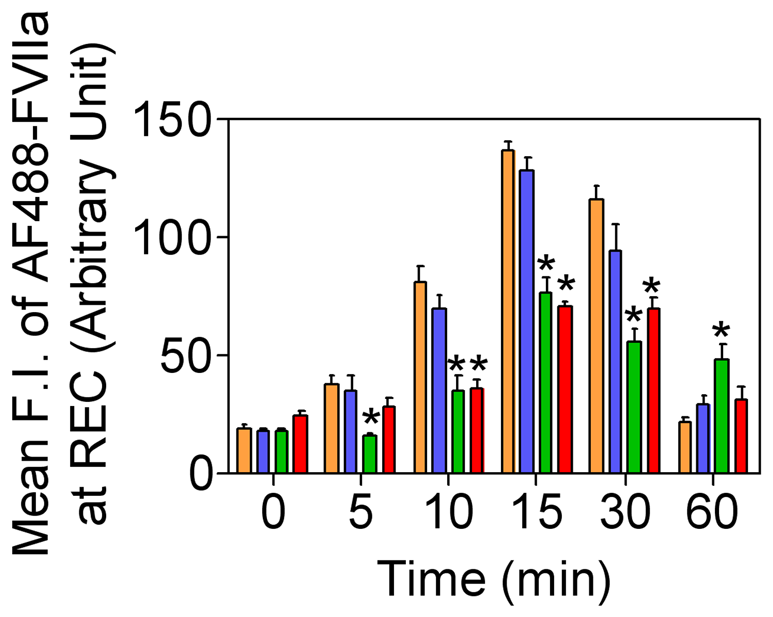
Accumulation of FVIIa in the recycling endosomal compartment in CHO-EPCR cells expressing Rab5A and its variants. CHO-EPCR cells transduced with control adenoviruses (brown) or recombinant adenoviruses encoding wild-type (blue), constitutively active (green), or dominant negative (red) Rab5A were incubated with AF488-FVIIa (50 nM) for 1 h at 4°C, and allowed to internalize for varying time periods at 37°C. FVIIa that accumulated in the REC was quantified by measuring the pixel density of the fluorescence of AF488-FVIIa in this compartment. * denotes that the value significantly differs from the values obtained in cells expressing endogenous Rab5A or wild-type Rab5A (*P<0.01*).

**Figure 6:**
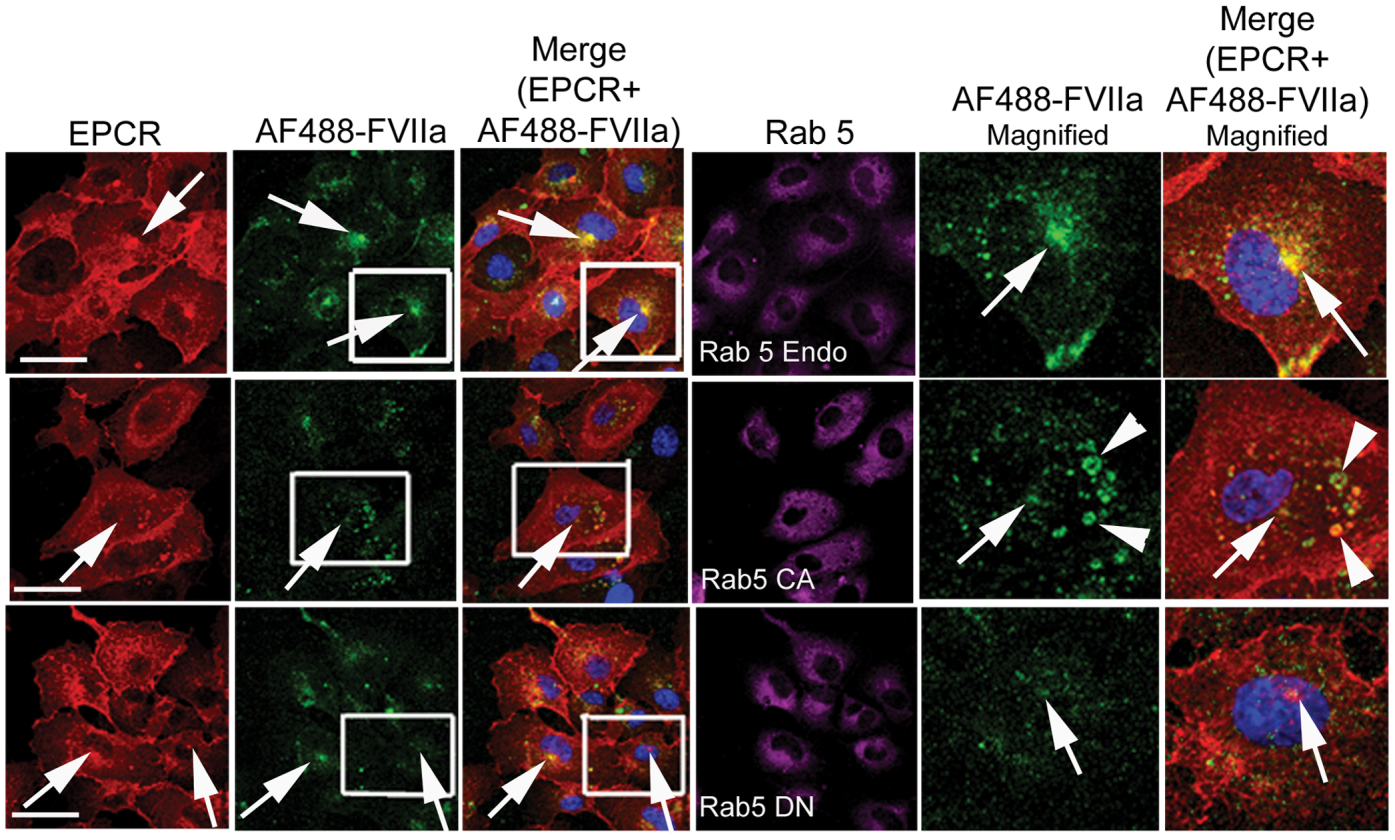
Rab 5A regulates FVIIa routing to the recycling endosomal compartment in endothelial cells. HUVEC transduced with control adenovirus or recombinant adenoviruses to express constitutively active or dominant negative Rab5A variants were incubated with AF488-FVIIa (50 nM) for 30 min at 37°C. Permeabilized cells were immunostained for EPCR and Rab5A, and imaged to localize EPCR, Rab5A, and AF488-FVIIa. The last two panels are the digitally enlarged images of the insets in 2^nd^ and 3^rd^ panels. Arrows indicate the accumulation of AF488-FVIIa in the REC. Therrowhead indicates entrapment of the ligand in abnormally enlarged early endosomal structures. The bar shown on images of HUVEC in this and other figures represents 20 µm length.

FVIIa internalization and trafficking in CHO-EPCR cells expressing WT Rab5A was very similar to that was observed with control CHO-EPCR cells, with the exception that endosomal structures were slightly larger in cells transduced with WT Rab5A (data not shown). It has been shown that overexpression of Rab5 WT or Rab5 CA mutants led to the formation of enlarged early endosomal structures due to the enhanced endosome-endosome fusion mediated by GTP bound Rab5 [27]–[29]. In agreement with these reports, CHO-EPCR cells overexpressing Rab5A CA mutant exhibited enlarged early endosomal structures, which were stained positively with Rab5 antibody (Fig. 3). The internalized AF488-FVIIa was found to be localized in these enlarged endosomal structures at all time points, even after 1 h of internalization (Fig. 3). Accumulation of AF488-FVIIa in the REC at 15 and 30 min was diminished, indicating that Rab5A CA mutant partly inhibited the trafficking of AF488-FVIIa from early endosome to the REC (Fig. 3). The colocalization of AF488-FVIIa and EPCR in these enlarged endosomal structures indicate that EPCR and FVIIa exist as a complex trapped within this compartment. In cells expressing DN Rab5A^S34N^, very few vesicular structures containing AF488-FVIIa and EPCR were seen beneath the plasma membrane, suggesting that expression of DN Rab5A inhibited the endocytosis of EPCR-FVIIa complexes (Fig. 4). The impaired internalization of FVIIa resulted in a lower accumulation of AF488-FVIIa at the pericentriolar REC in cells expressing Rab5A^S34N^ (Fig. 5). As observed in CHO-EPCR cells, large endosomal structures containing AF488-FVIIa and EPCR were also found in HUVEC overexpressing Rab5A CA mutant that led to the decreased accumulation of AF488-FVIIa and EPCR at the REC in these cells (Fig. 6). HUVEC overexpressing Rab5A DN mutant showed less internalization of AF488-FVIIa as compared in control cells (Fig. 6).

### Rab4A Regulates Recycling of AF488-FVIIa from Early/sorting Endosomes

**Figure 7:**
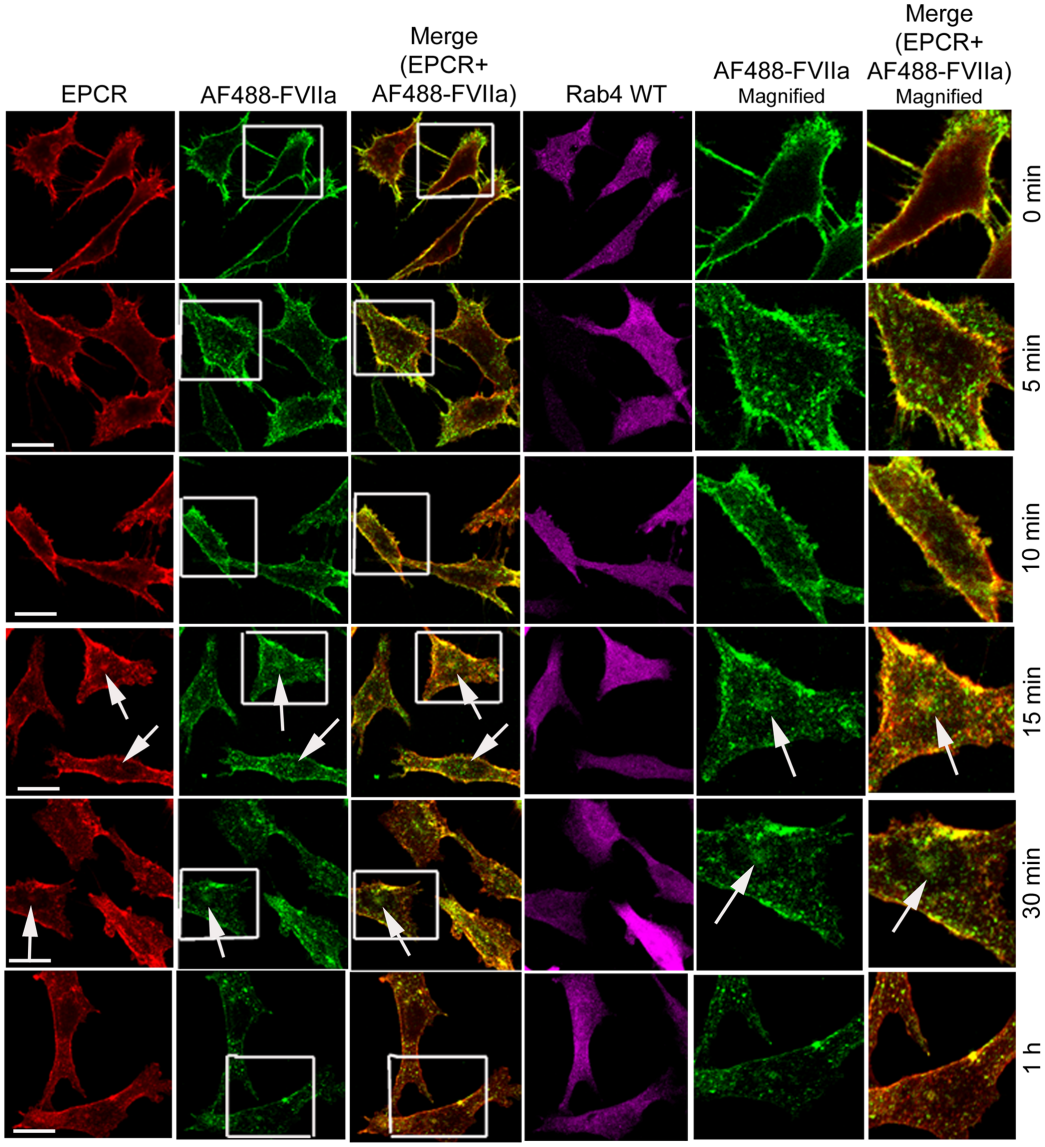
Overexpression Rab4A impairs trafficking of FVIIa from early endosomes to the recycling compartment. CHO-EPCR cells were transduced with adenoviruses encoding wild-type Rab4A. The transduced cells were incubated with AF488-FVIIa (50 nM) for 1 h at 4°C, and then transferred to 37°C to induce internalization of the surface bound ligand. The permeabilized cells were immunostained for EPCR and Rab4A, and immunofluorescence and fluorescence of AF488-FVIIa were analyzed by confocal microscopy. The two right two panels are the digitally enlarged images of the insets of 2^nd^ and 3^rd^ panels to clearly illustrate differences in the intracellular localization of FVIIa at different time intervals. The arrow indicates the accumulation of AF488-FVIIa in the REC.

**Figure 8:**
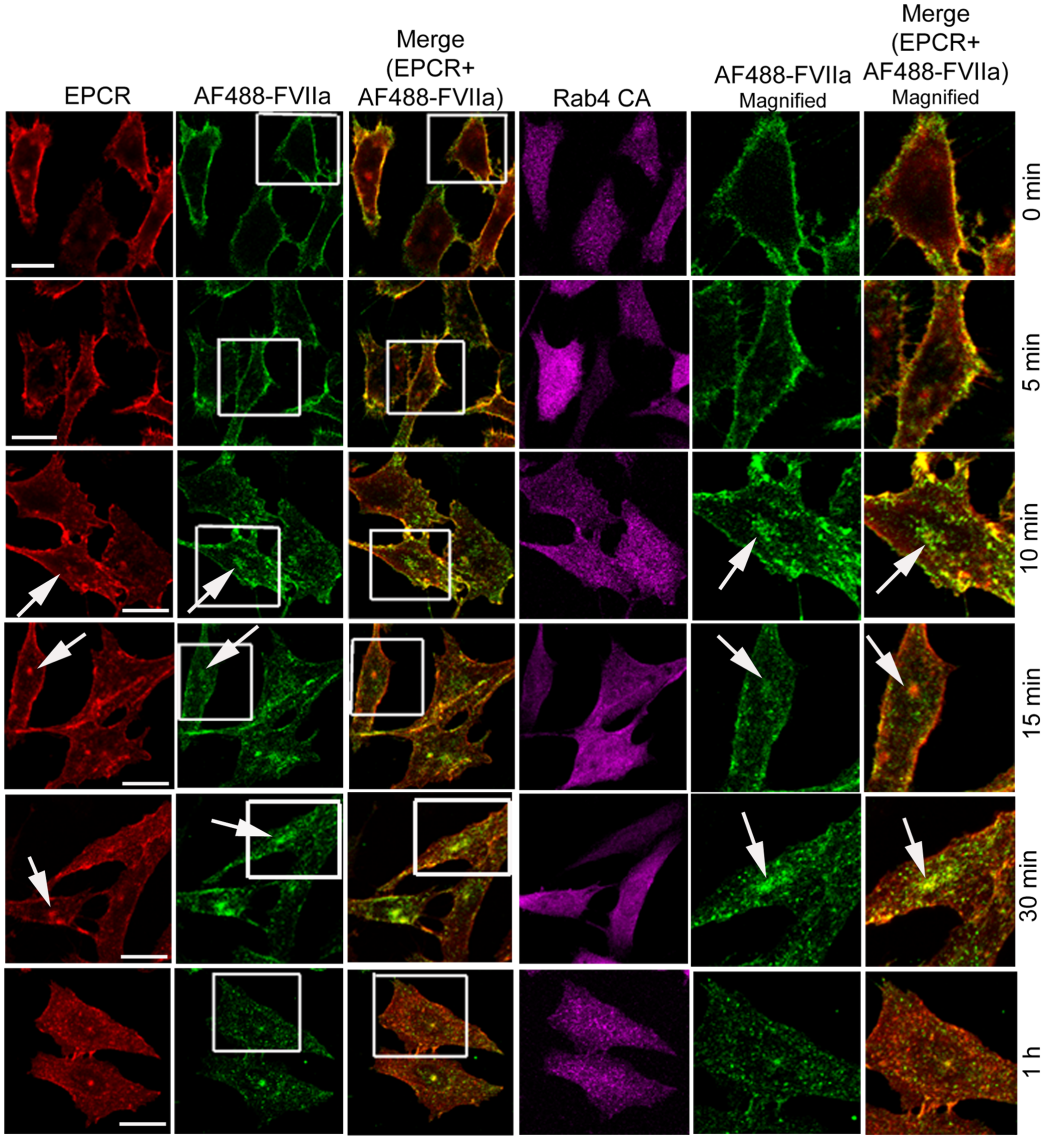
Effect of overexpression of constitutively active Rab4A mutant on intracellular trafficking of FVIIa and EPCR. The experimental procedure and the image acquisition were essentially the same as described in Fig. 7 except that CHO-EPCR cells were transduced with adenoviruses encoding constitutively active Rab4A, instead of wild-type Rab4A.

**Figure 9:**
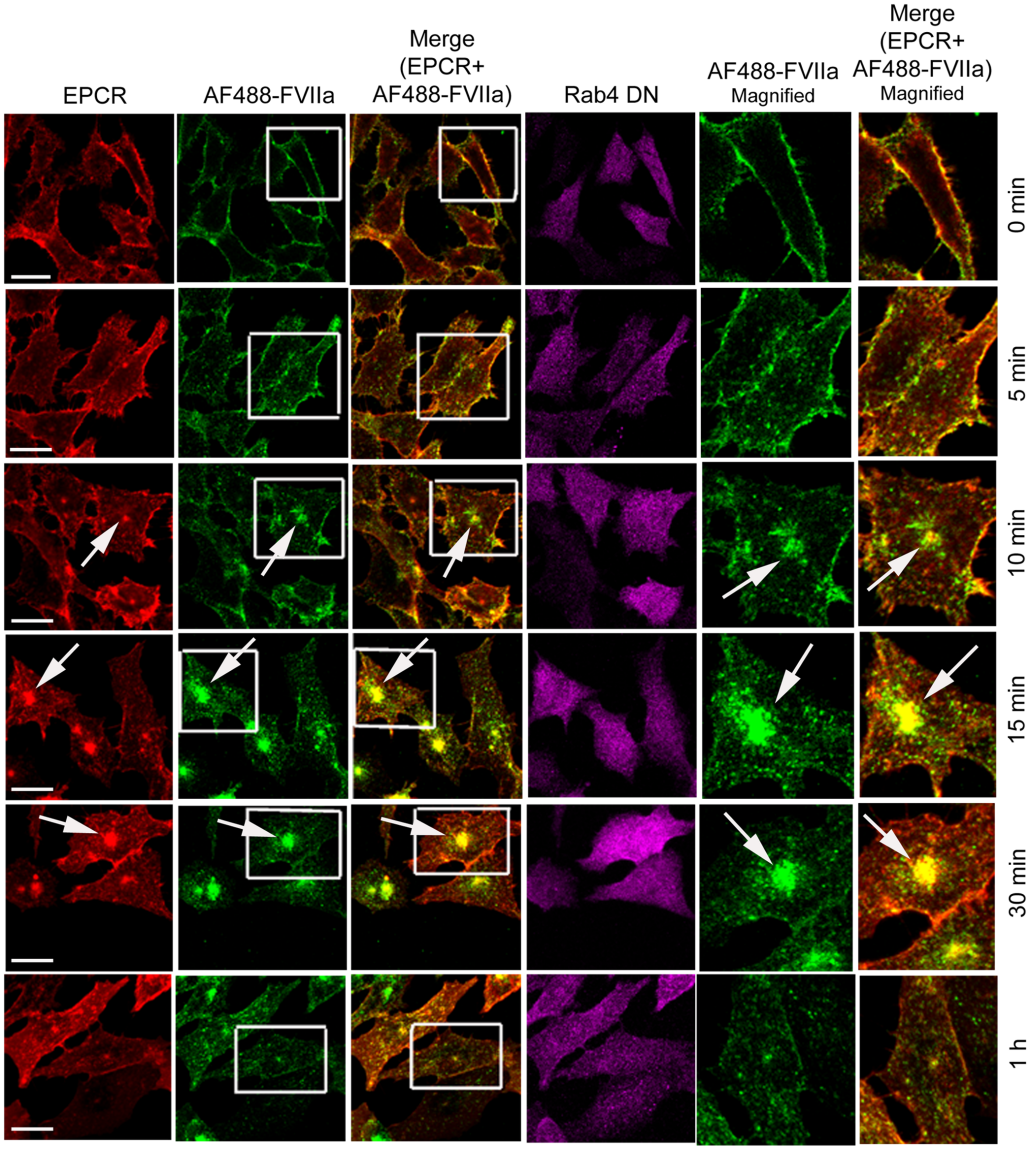
Effect of overexpression of dominant negative mutant of Rab4A on intracellular trafficking of FVIIa and EPCR. The experimental procedure is essentially the same as described in Fig. 7 except that CHO-EPCR cells were transduced with adenoviruses encoding dominant negative mutant of Rab4A, instead of wild-type Rab4A.

**Figure 10:**
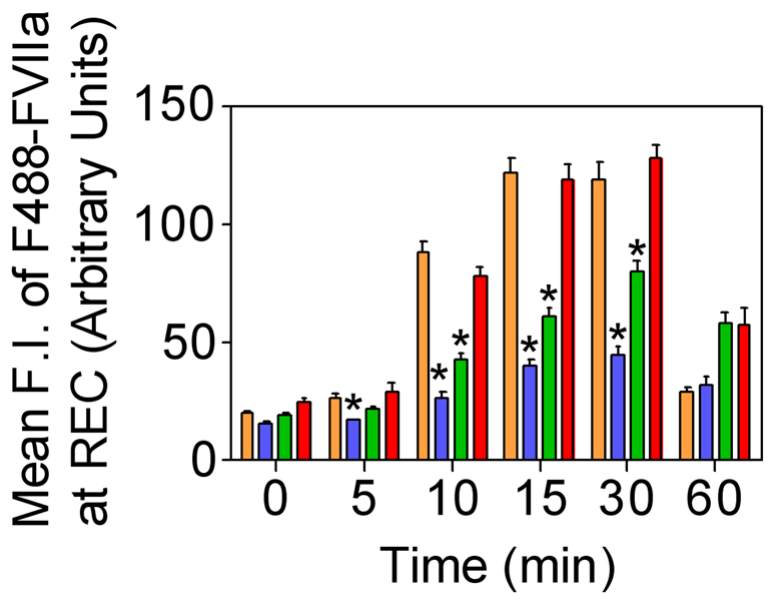
Effect of overexpression of wild-type, constitutively active, or dominant negative variant of Rab4A on accumulation of FVIIa in the recycling endosomal compartment. CHO-EPCR cells transduced with control adenoviruses (brown) or recombinant adenoviruses encoding wild-type (blue), constitutively active (green), or dominant negative (red) Rab4A were incubated with AF488-FVIIa (50 nM) for 1 h at 4°C, and allowed to internalize for varying time periods at 37°C. FVIIa accumulated in the REC was quantified by measuring the pixel density of the fluorescence of AF488-FVIIa in this compartment. * denotes that the value significantly differs from the values obtained in cells expressing endogenous Rab4A (*P<0.01*).

**Figure 11:**
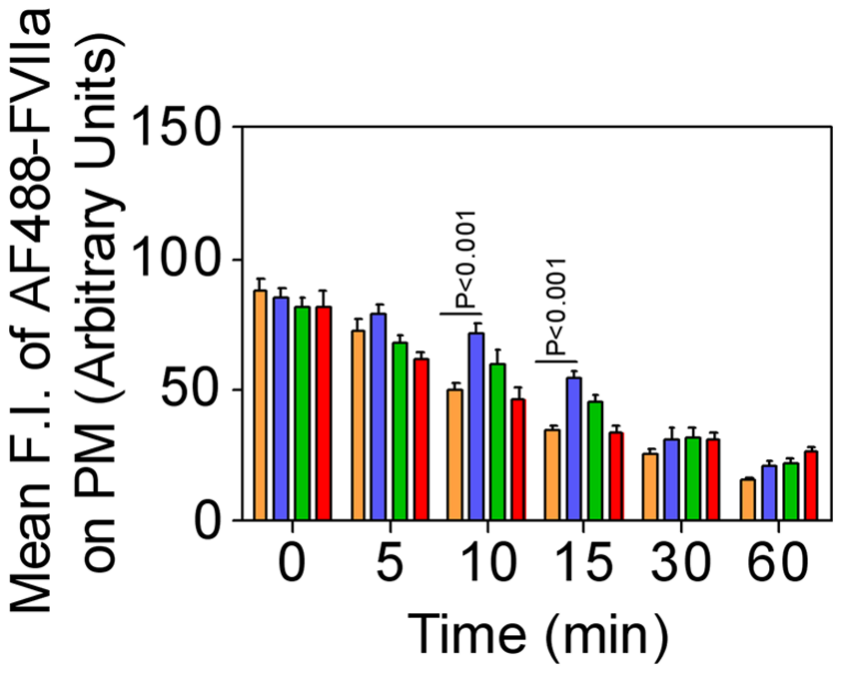
Increased recycling of endocytosed FVIIa to the plasma membrane in CHO-EPCR cells overexpressing wild-type Rab4A. CHO-EPCR cells transduced with control adenoviruses (brown) or recombinant adenoviruses encoding wild-type (blue), constitutively active (green), or dominant negative (red) Rab4A were incubated with AF488-FVIIa (50 nM) for 1 h at 4°C, and allowed to internalize for varying time periods at 37°C. The recycling of the ligand to the cell surface was quantified by measuring the pixel intensity of AF488-FVIIa on the plasma membrane at different time intervals.

Rab4A is known to play a critical role in the recycling of receptor/ligand complexes from early/sorting endosomes back to the cell surface. Overexpression of WT Rab4A in CHO-EPCR cells impaired trafficking of AF488-FVIIa from early endosomes to the REC as significantly less ligand accumulation was found at the REC in these cells even after 15 to 30 min following the onset of internalization (Fig. 7). EPCR trafficking to the REC is also inhibited in these cells as very little EPCR was found in this compartment compared to control cells (Fig. 7). The accumulation of AF488-FVIIa and EPCR in the REC was also substantially lower in CHO-EPCR cells expressing CA Rab4A^Q67L^ but not as severe as the levels observed in CHO-EPCR cells overexpressing WT Rab4A (Fig. 8). CHO-EPCR cells expressing DN Rab4A^S34N^ showed a similar or slightly increased level of FVIIa and EPCR accumulation in the REC as that which was observed in control CHO-EPCR cells (Fig. 9). The mean fluorescence intensity of AF488-FVIIa within the REC in WT Rab4A and CA Rab4A expressing cells was significantly lower than in cells expressing Rab4A endogenously or transfected to express DN Rab4A (Fig. 10). To identify the role of Rab 4 in recycling of the ligand from early/sorting endosome back to the cell surfaces, we measured the mean fluorescence intensity of AF488-FVIIa on the plasma membrane (PM) at various time points after the onset of internalization in control CHO-EPCR cells and CHO-EPCR cells overexpressing Rab4A WT, CA, or DN variants. The mean fluorescence intensity of AF488-FVIIa on the PM, at 10 and 15 min time points, was significantly higher in CHO-EPCR cells overexpressing Rab4A WT compared to control or Rab4A DN expressing CHO-EPCR cells (Fig. 11). These data suggest an increased recycling of the ligand from early/sorting endosomes back to the cell surface in Rab4A WT overexpressing cells, which resulted in decreased trafficking of the ligand from early endosomes to the REC in these cells.

**Figure 12:**
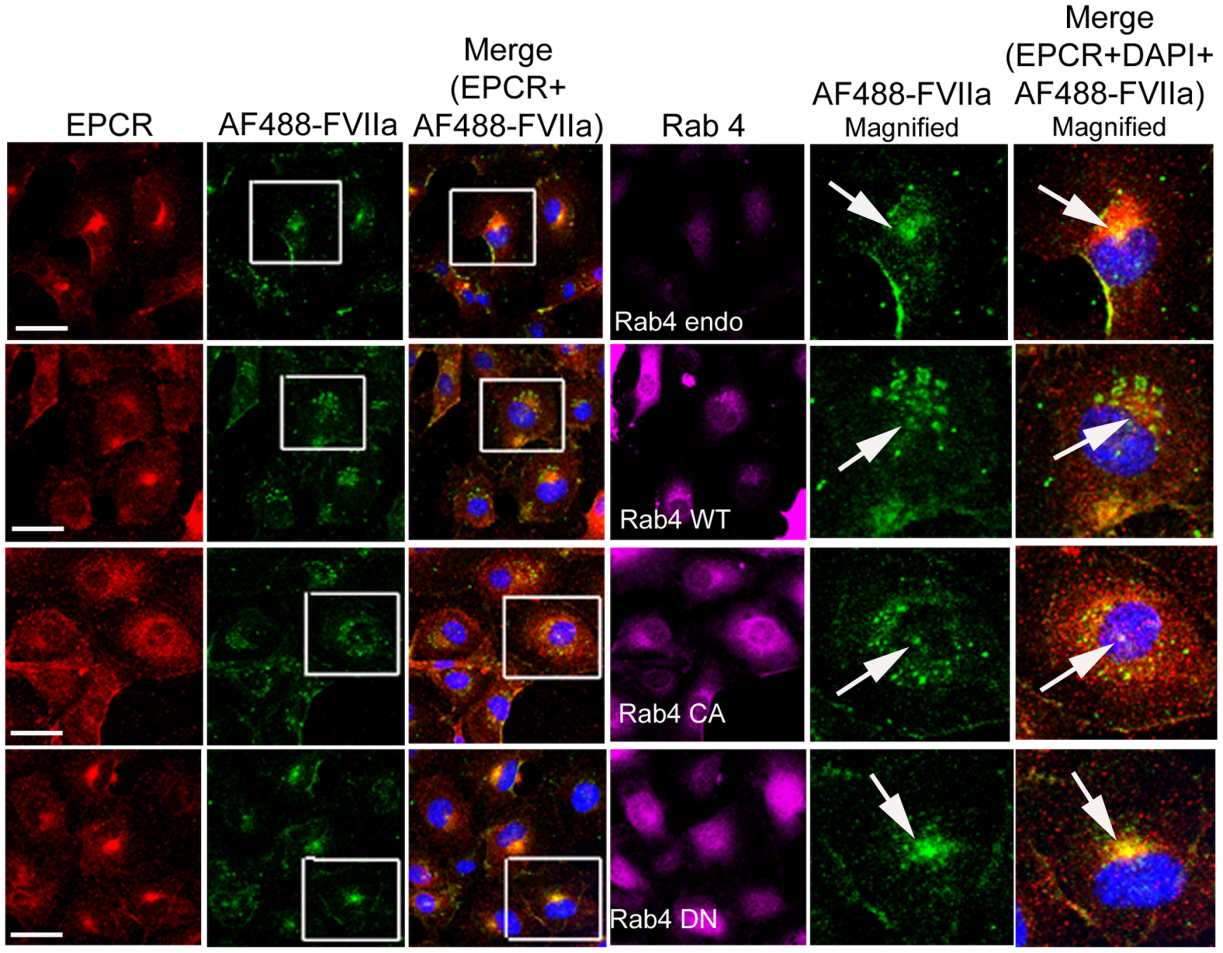
The role of Rab4A in intracellular trafficking of FVIIa in endothelial cells. HUVEC transduced to express wild-type, constitutively active, or dominant negative Rab4A variants were incubated with AF488-FVIIa (50 nM) for 30 min at 37°C. Then, the cells were fixed, permeabilized, immunostained for EPCR and Rab4, and imaged to localize EPCR, Rab4, and AF488-FVIIa. Arrows mark the presence of AF488-FVIIa and EPCR in REC at the juxtanuclear position.

We also analyzed the steady state internalization of AF488-FVIIa in HUVEC transduced to overexpress Rab4A WT, CA, or DN variants. Similar to that observed in CHO-EPCR cells, the trafficking of AF488-FVIIa from early endosome to the REC was impaired in HUVEC expressing WT or CA mutant, and therefore the amount of the ligand accumulated at the REC was lower in these cells compared to control cells (Fig. 12). HUVEC overexpressing Rab4A DN mutant showed a similar or slightly increased level of AF488-FVIIa accumulation at the REC.

### Rab11 Regulates Recycling of Internalized FVIIa to and from the Recycle Endosomal Compartment

**Figure 13:**
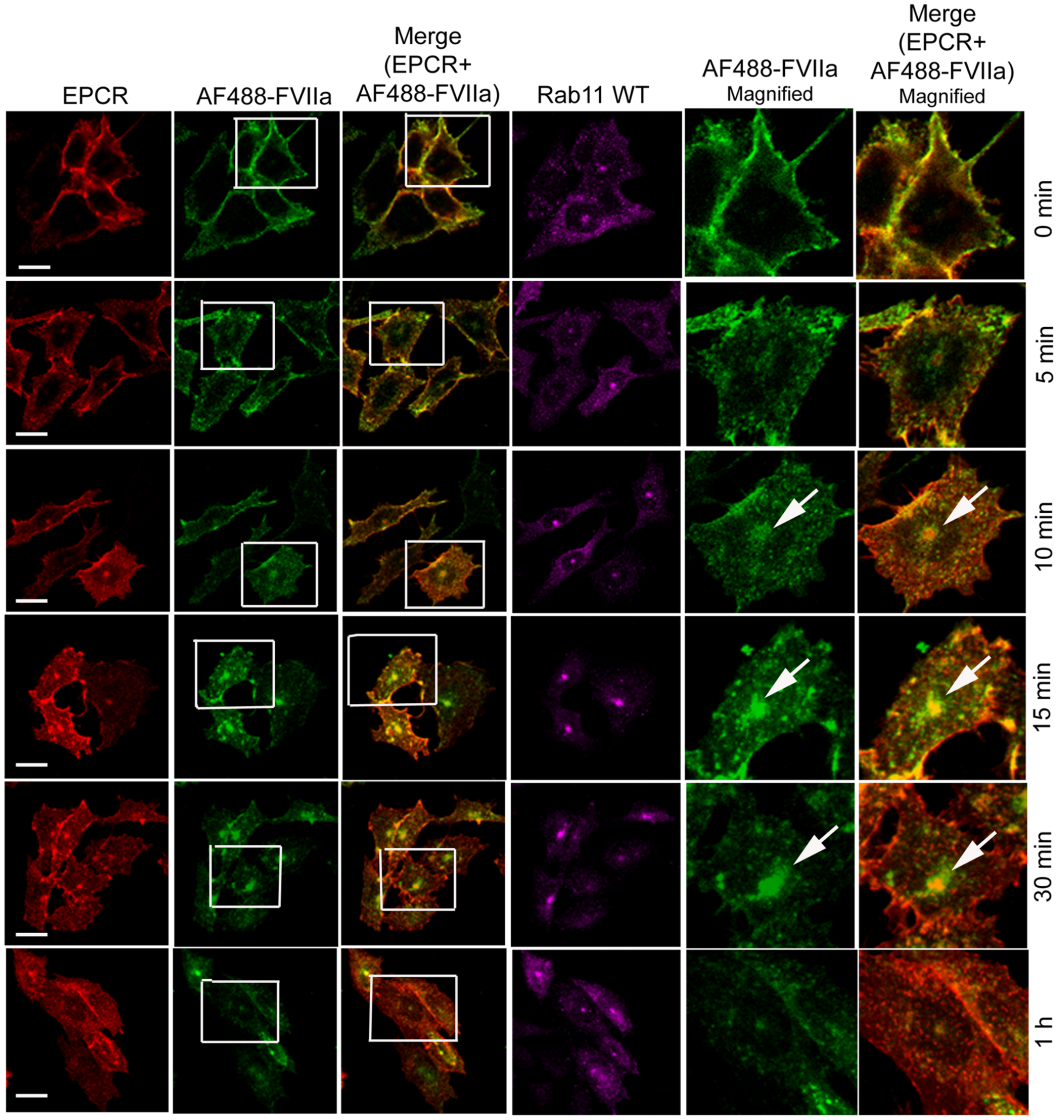
Overexpression of wild-type Rab11 does not significantly affect of EPCR-dependent FVIIa endocytosis and trafficking. CHO-EPCR cells transduced with adenovirus encoding wild-type Rab11 were exposed to AF488-FVIIa (50 nM) for 1 h at 4°C. Then, the unbound ligand was removed and the cells were transferred to 37°C to induce internalization of the surface bound ligand. After varying times at 37°C, the cells were fixed, permeabilized and immunostained for EPCR and Rab11. The cells were imaged for immunofluorescence of EPCR and Rab11, and fluorescence of AF488-FVIIa. The two right panels are digitally enlarged images of insets of 2^nd^ and 3^rd^ panels, respectively, to provide a better illustration of differences in the intracellular localization of FVIIa at varying time intervals. Arrows indicate the accumulation of AF488-FVIIa and EPCR in the REC at the juxtanuclear region.

**Figure 14:**
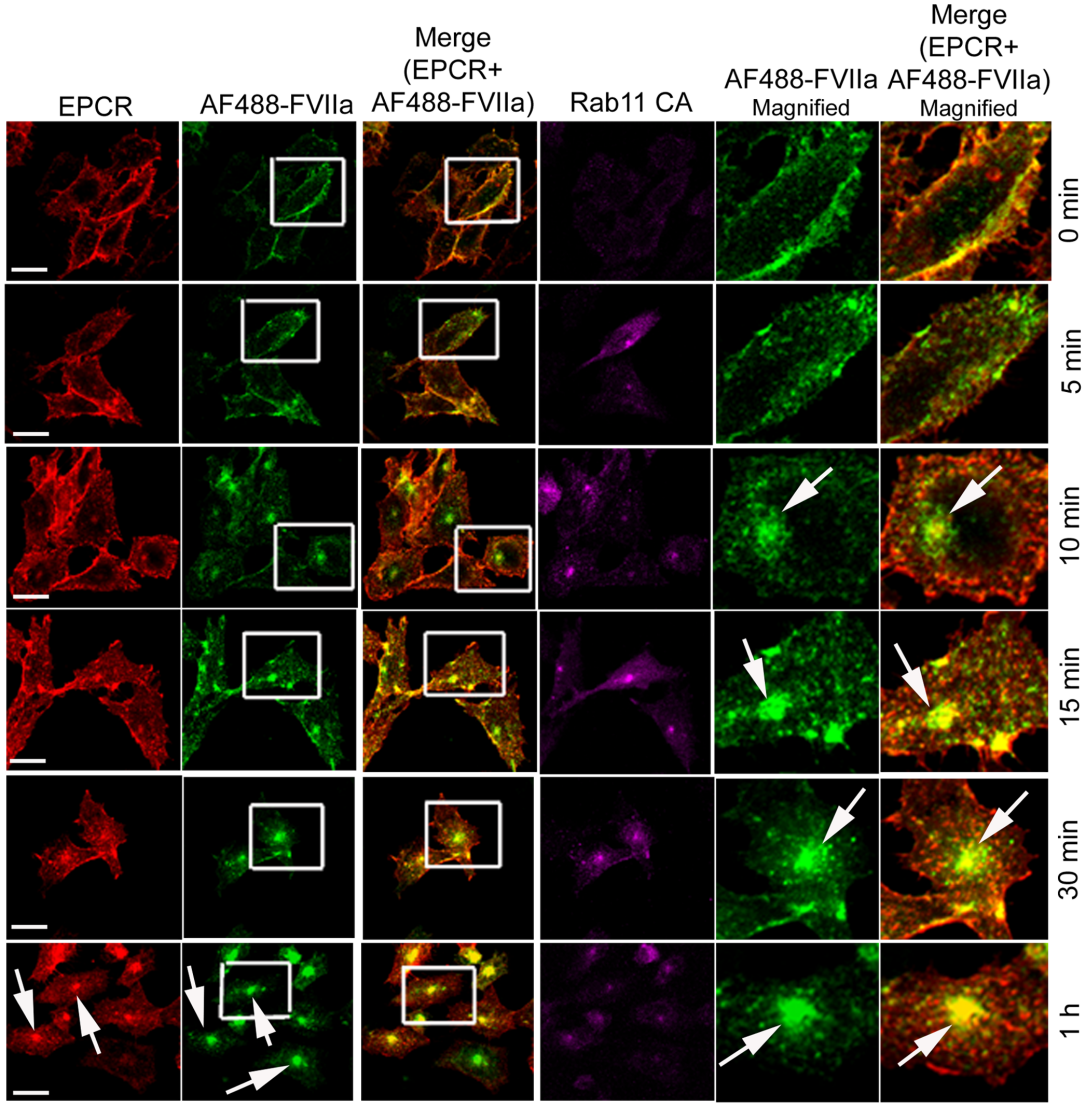
Overexpression of constitutively active Rab11 impairs the recycling of FVIIa and EPCR from the recycling compartment. The experimental procedure and the image acquisition were essentially the same as described in Fig. 13 except that CHO-EPCR cells were transduced with adenoviruses encoding constitutively active Rab11, instead of wild-type Rab11.

**Figure 15:**
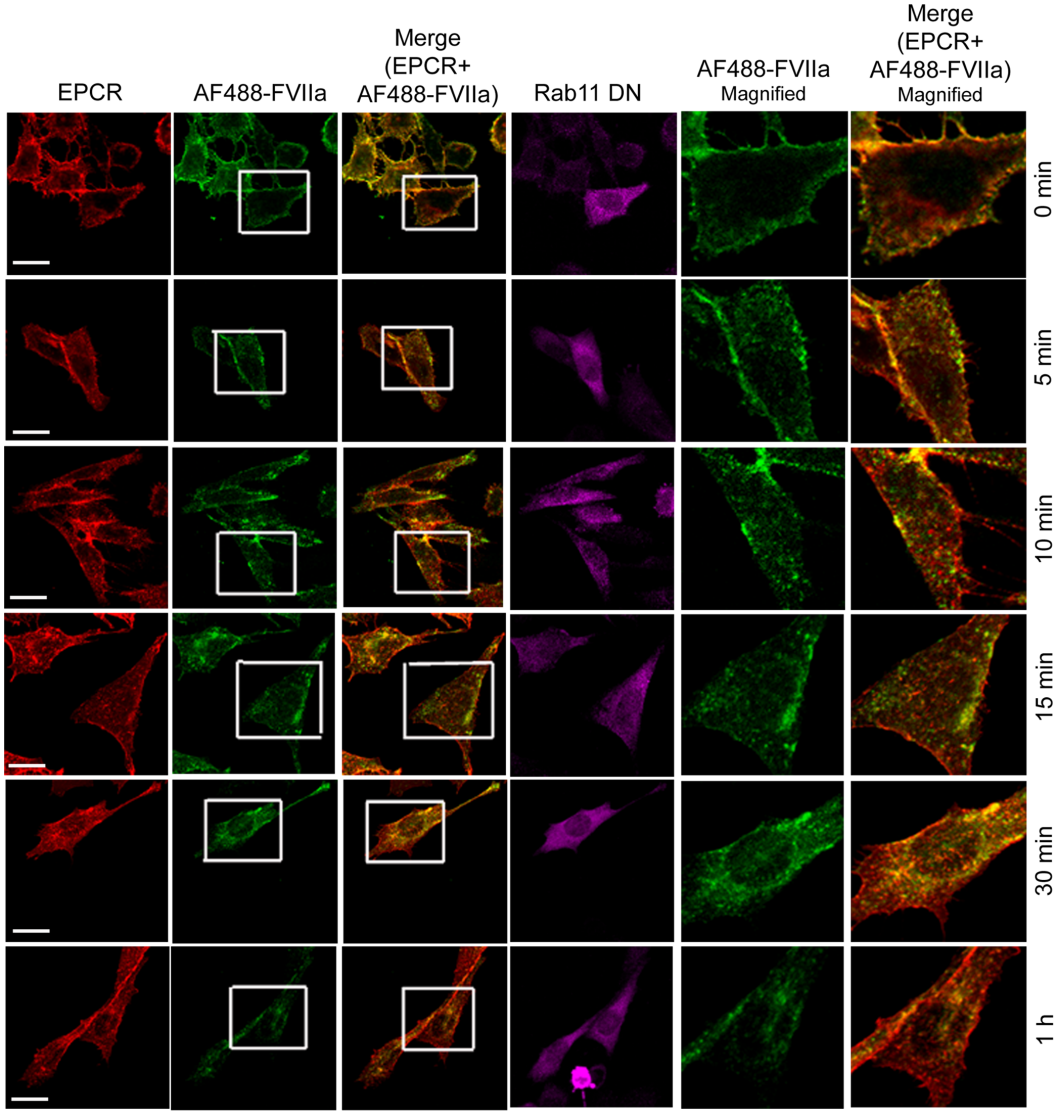
Overexpression of dominant negative variant of Rab11 inhibits the entry of internalized FVIIa and EPCR into the recycling compartment. The experimental procedure and the image acquisition were essentially the same as described in Fig. 13 except that CHO-EPCR cells were transduced with adenoviruses encoding dominant negative mutant of Rab11, instead of wild-type Rab11.

**Figure 16:**
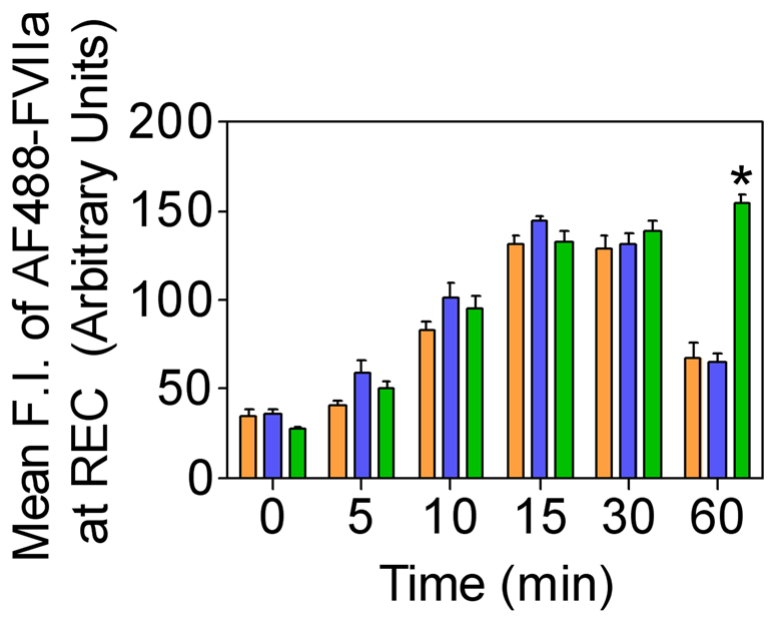
Overexpression of Rab11 retains the internalized FVIIa in the recycling endosomal compartment. CHO-EPCR expressing endogenous Rab11 (brown) or CHO-EPCR cells transduced to overexpress wild-type Rab11 (blue) or constitutively active Rab11 (green) were incubated with AF488-FVIIa (50 nM) for 1 h at 4°C, and allowed to internalize for varying time periods at 37°C. FVIIa accumulated at varying times in the REC was quantified by measuring the pixel density of the fluorescence of AF488-FVIIa in this compartment. * denotes that the value significantly differs from the values obtained in cells expressing endogenous Rab11 or wild-type Rab11 (*P<0.01*).

**Figure 17:**
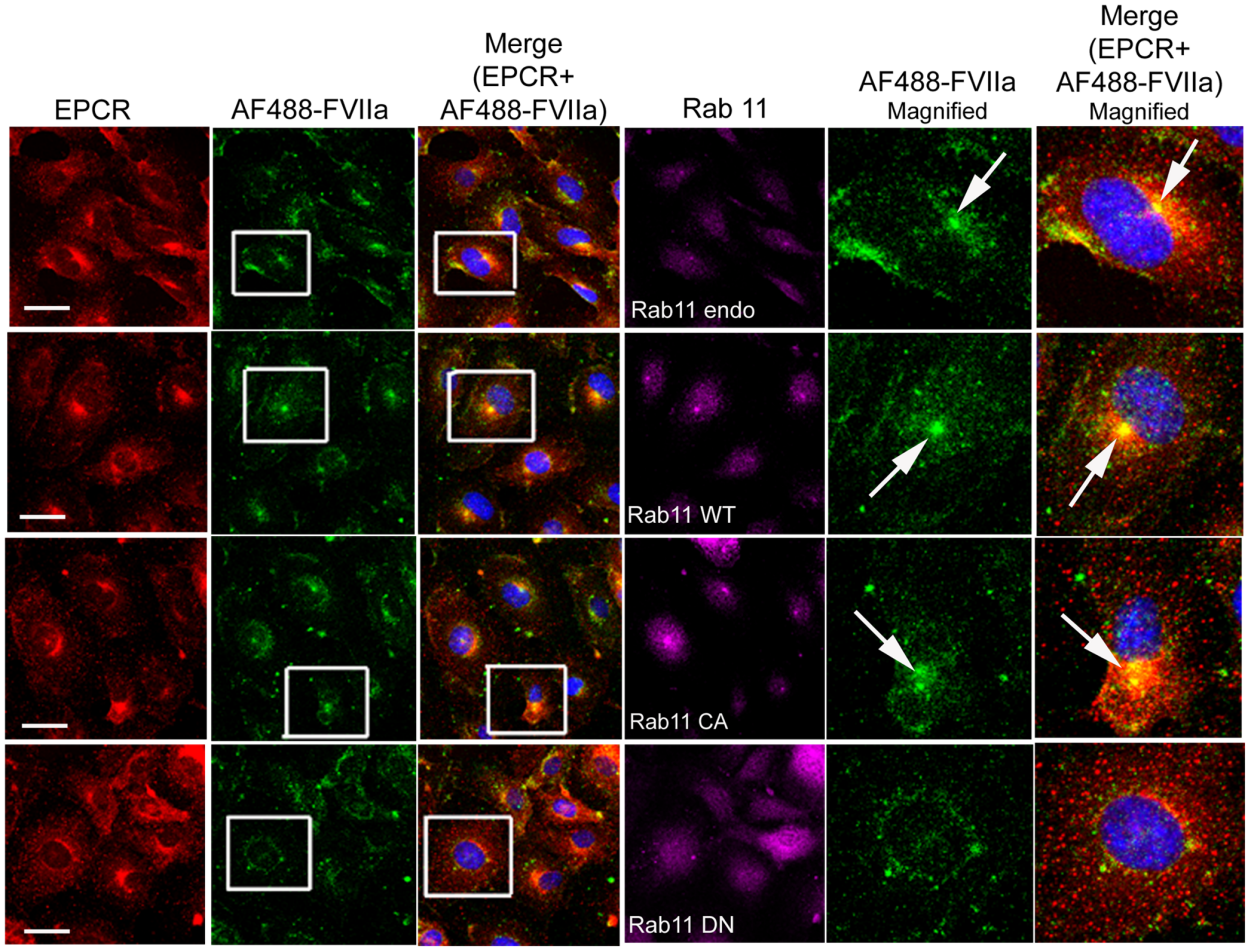
Effect of Rab11 GTPase on FVIIa trafficking in endothelial cells. HUVEC transduced with control or recombinant adenoviruses to express wild-type, constitutively active or dominant negative Rab11 were incubated with AF488-FVIIa (50 nM) for 30 min at 37°C. Then, the cells were fixed, permeabilized, and immuonstained for EPCR and Rab11, and imaged to observe localization of EPCR, Rab11 and AF488-FVIIa. The two right panels are digitally enlarged images of the insets of 2^nd^ and 3^rd^ panels, respectively. Arrows indicate the accumulation of AF488-FVIIa and EPCR in REC at the juxtanuclear region.

Rab11 has been shown to associate with the pericentriolar REC and play a critical role in the recycling of receptor/ligand complexes from this compartment back to the cell surface. Overexpression of WT Rab11 in CHO-EPCR cells showed no significant effect in EPCR-FVIIa trafficking (Fig. 13). In contrast, expression of CA Rab11^Q70L^ prevented the recycling of FVIIa and EPCR which had entered the REC, thus increasing the total accumulation of FVIIa and EPCR in the REC. Even 1 h after the onset of internalization, most of the internalized FVIIa was found in the REC of these cells (Fig. 14). In contrast to CA Rab11, expression of DN Rab11 (Rab11^S25N^) inhibited the entry of FVIIa and EPCR into the REC and thus the internalized ligand population was found to be distributed throughout the cytoplasm (Fig. 15). The mean fluorescence intensity of AF488-FVIIa within the REC of CA Rab11 expressing cells at 1 h after onset of internalization was significantly higher than that found in endogenously expressing or adenovirus transduced WT Rab11 cells (Fig. 16). Analysis of the steady state internalization of AF488-FVIIa in HUVEC transduced with Rab11 variants further confirmed that DN Rab11 inhibited the accumulation of internalized FVIIa in the REC (Fig. 17). Overall, these data suggest that Rab11 plays a key role in facilitating the trafficking of ligands from early endosomes to the REC at the juxtanuclear region and recycling from this compartment.

### Rab7 does not Appear to Influence FVIIa-EPCR Intracellular Trafficking

**Figure 18:**
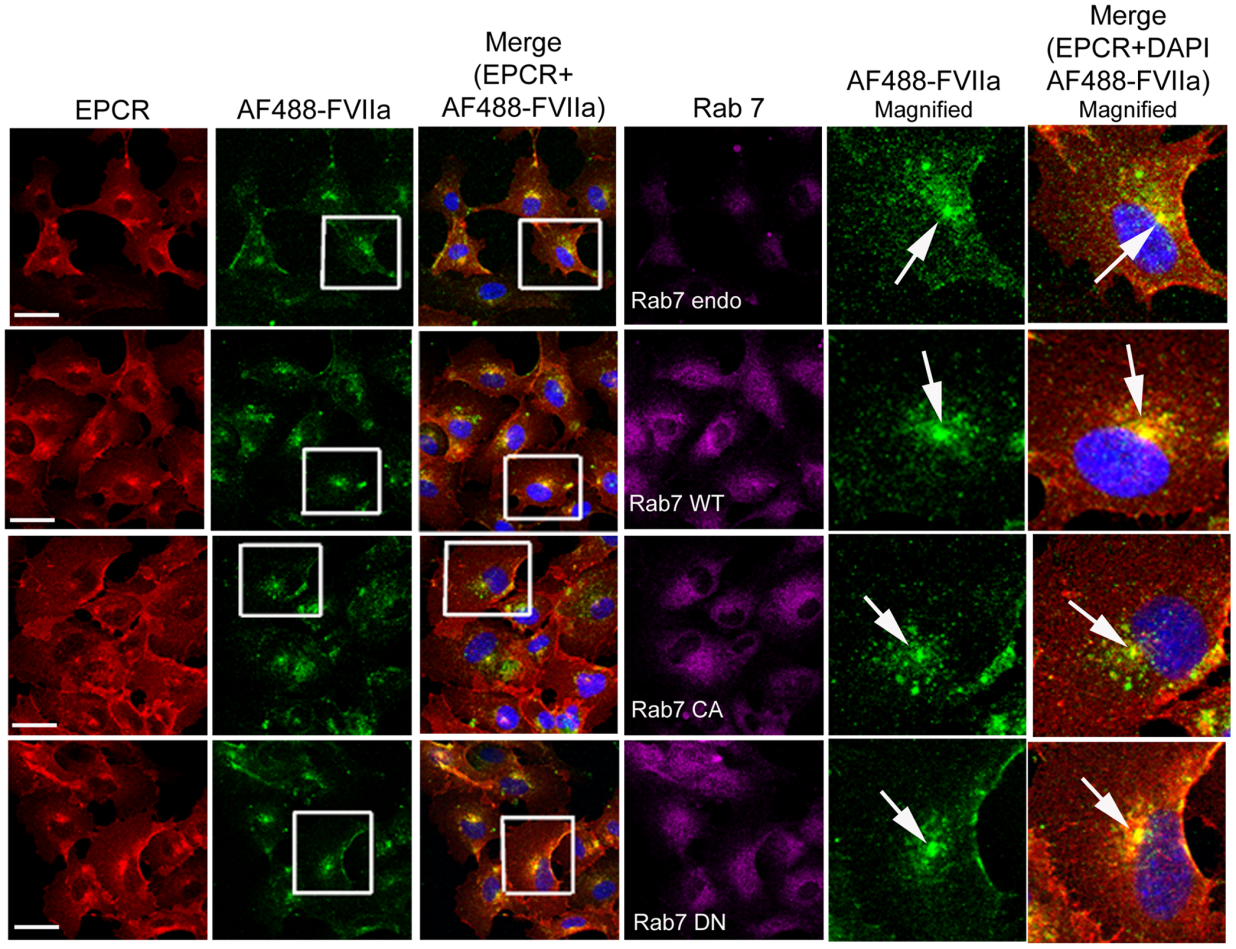
Rab 7 does not play a significant role in the intracellular trafficking of FVIIa. HUVEC transduced with control or recombinant adenoviruses to express wild-type, constitutively active or dominant negative Rab7 were incubated with AF488-FVIIa (50 nM) for 30 min at 37°C. Then, the cells were fixed, permeabilized, and immuonstained for EPCR and Rab7, and imaged to observe localization of EPCR, Rab7, and AF488-FVIIa. Right two panels are digitally enlarged images of the insets of 2^nd^ and 3^rd^ panels. Arrow indicates the accumulation of AF488-FVIIa and EPCR at REC at the juxtanuclear region.

In additional studies, we investigated the effect of overexpression of WT, CA, and DN Rab7 variants on EPCR-FVIIa endocytosis and trafficking. No noticeable differences were observed in EPCR-FVIIa endocytosis or intracellular trafficking of internalized EPCR and FVIIa in HUVEC overexpressing Rab7 WT, CA or DN variants (Fig. 18). The pattern of FVIIa trafficking in CHO-EPCR cells overexpressing Rab7 mutants was very similar to that which was observed in non-transfected CHO-EPCR cells (data not shown), confirming that Rab7 may not play an important role in determining the fate of internalized FVII-EPCR complex.

### Rab 11 Plays a Role in FVIIa Transcytosis

**Figure 19:**
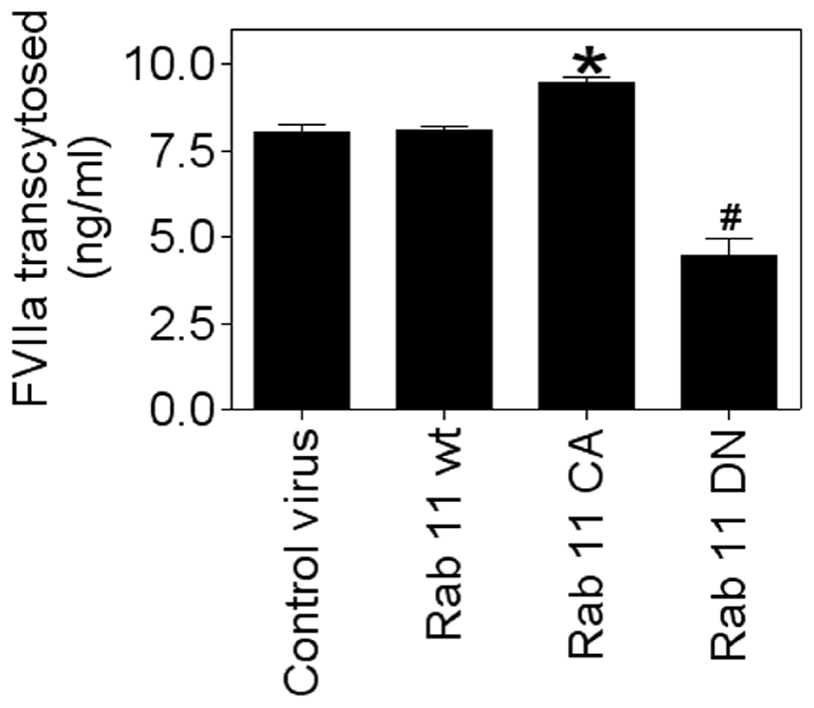
Involvement of Rab11 GTPase in FVIIa transport from the apical to basal side. HUVEC cultured in transwells were infected with control adenovirus or adenoviruses encoding wild-type Rab11 or Rab11 variants (20 moi/cell), and grown to reach full confluency. Then, FVIIa (10 nM) was added to the upper chamber. Two hours after adding FVIIa, the medium from the bottom chamber was removed and measured for FVIIa activity levels in factor X activation assay. The data shown in the figure represent mean ± SEM from 3 to 6 independent experiments. *indicates a statistical significant difference from the value obtained with HUVEC infected with control virus (*P*<0.01); ^#^, differs in a statistically significantly manner from all other values shown in the figure (*P*<0.001).

Since Rab11 was shown to regulate transcytotic migration of internalized ligands from apical to basal surfaces in polarized epithelial cells [21] and our earlier studies suggested that EPCR may facilitate transcytosis of FVIIa [3], we investigated the effect of Rab11 on FVIIa transcytosis. As shown in Fig. 19, the overexpression of WT Rab11 had no significant effect on FVIIa transcytosis whereas overexpression of CA Rab11 slightly but statistically significantly increased FVIIa transcytosis. In contrast, overexpression of DN Rab11 markedly inhibited the transport of FVIIa from the apical to basal surface.

### Radioligand Studies

To further strengthen the above data obtained from microscopic studies, we attempted to quantify, more objectively, differences in the endocytosis and recycling of FVIIa and EPCR following expression of various Rab GTPases and their variants by monitoring FVIIa and EPCR using^125^I-labeled FVIIa and EPCR mAb. However, these studies failed to yield robust and conclusive data. A probable explanation for this is that small amounts of FVIIa and EPCR endocytosed and trafficked would be much easier to detect by confocal microscopy as they will have targeted organization into distinctive membrane compartments. Furthermore, basal non-specific/EPCR-independent binding and internalization does not interfere in image analysis as they do not result in targeted organization and dense accumulations. Internalization and recycling assays using radiolabeled ligands measure global differences, and thus it may be difficult to capture distinctive differences in intracellular trafficking mediated by Rab GTPase variants using these methods.

## Discussion

Our recent studies showed that FVIIa binding to EPCR promoted the endocytosis of EPCR via dynamin and caveolar-dependent pathways and the endocytosed receptor-ligand complexes accumulated in the REC before being targeted back to the cell surface [3]. EPCR-mediated FVIIa endocytosis/recycling appears to play a role in the transport of FVIIa from the apical to basal cell surface [3]. Our recent studies indicate that EPCR plays a critical role in transporting FVIIa from circulation to extravascular tissues [30]. Rab GTPases (Rab4, Rab5, Rab7 and Rab11 etc.), which localize to specific endosomal structures, have been shown to play crucial roles in the endocytic and exocytic pathways of receptor or receptor/ligand complexes. In the present study, we investigated the role of different Rab GTPases on EPCR-FVIIa endocytosis and the intracellular trafficking of endocytosed FVIIa and EPCR by overexpressing the wild-type, constitutively active, or dominant negative Rab GTPase variants in endothelial and CHO-EPCR cells. The data presented herein show that Rab GTPases regulate the endocytosis and intracellular trafficking of EPCR and endocytosed FVIIa.

Colocalization analyses of the internalized FVIIa with various Rab proteins (Rab5, Rab4, Rab11 and Rab7) at different time intervals following FVIIa internalization showed that immediately following internalization (at 5 min), FVIIa colocalizes extensively with Rab5 positive endosomes, suggesting entry of internalized FVIIa into these early endosomal compartments. At this early time, internalized FVIIa also colocalizes with Rab4 positive endosomal structures, indicating targeted trafficking of the internalized ligand into the sorting endosomes as well. However, the colocalization efficiency between Rab4 and FVIIa is lower than that observed between Rab5 and FVIIa. These data suggest that some but not all of the internalized FVIIa is sorted to Rab4 positive sorting endosomes. As expected from our earlier study [3], the internalized FVIIa colocalizes with Rab11 after 15 min following internalization, indicating entry of ligand into the pericentriolar REC. We did not detect any appreciable colocalization of the internalized ligand with Rab7, a Rab protein that regulates protein trafficking in the endocytic pathway from early endosomes to late endosomal structures, which routes the endocytosed receptors and/or ligands to lysosomes for degradation [31]. This suggests that most of the internalized FVIIa and EPCR may escape lysosomal degradation.

Rab5 localizes to the plasma membrane and early endosomal structures, and plays a critical role in the endocytosis of receptor and receptor/ligand complexes from the plasma membrane to the early endosomes [11], [32]. Rab5 has also been shown to facilitate the homotypic fusion between early endosomes as the expression of constitutively active Rab5 was found to lead to the formation of enlarged early endosomal structures, resulting from enhanced fusion between the endosomes [29]. Consistent with this, we observed enlarged endosomal structures beneath the plasma membrane in both HUVEC and CHO-EPCR cells transduced to express the constitutively active Rab5 mutant. Following the onset of internalization, FVIIa and EPCR were found to be accumulated in these Rab5 positive, enlarged endosomes. Expression of constitutively active Rab5 reduced the accumulation of FVIIa at the pericentriolar REC as FVIIa in the enlarged endosomes was not trafficked efficiently to the REC. Retention of FVIIa in the enlarged early endosomes even 1 h after its internalization suggests that transport of the internalized EPCR-FVIIa complexes from the early endosomes to the sorting endosomes is also impaired in cells expressing constitutively active Rab5. In cells expressing the dominant negative Rab5 mutant, very little FVIIa was found in the endosomes at early time points following the induction of FVIIa internalization, which suggests that expression of the Rab5 dominant negative mutant inhibited the endocytosis of EPCR-FVIIa. Overall, these data suggest that Rab5 plays a crucial role in the internalization of EPCR-FVIIa complexes from the plasma membrane and their entry into early endosomal structures of the endocytic pathway.

Rab4 has been shown to play a vital role in the recycling of receptor or ligand from sorting endosomes back to the cell surface [15]. The recycling of many G-protein coupled receptors, i.e. β2-adrenergic receptor, neurokinin 1 receptor, and CB1 cannabinoid receptor, is regulated by Rab4 [12], [33]. In HL-1 cardiac myocytes, transient expression of Rab4 GTPase facilitated the recycling of internalized β- adrenergic receptor and enhanced its signaling [34]. Overexpression of Rab4 GTPase in the present study dramatically attenuated the accumulation of internalized FVIIa and EPCR in the REC, and also increased the level of ligand recycling to the plasma membrane. This indicates, albeit indirectly, enhanced recycling of FVIIa from sorting endosomes back to the cell surface as a result of the overexpression of Rab4 GTPase, and thus further trafficking of the internalized FVIIa from early endosomes to the REC is reduced in these cells. Expression of Rab4 dominant negative mutant increased the accumulation of FVIIa and EPCR in the REC by inhibiting the recycling of receptor or ligand from early/sorting endosome back to cell surface. However, this increase was more modest than expected, suggesting that the trafficking of FVIIa and EPCR from early endosome to the REC may also be regulated by other factors.

When we examined the role of Rab11 in EPCR-FVIIa trafficking, we found that the expression of the dominant negative mutant Rab11 resulted in accumulation of FVIIa throughout the cytoplasm and very little in the REC. This suggests that Rab11 regulates the trafficking of FVIIa and EPCR from the early endosome to the REC. Interestingly, the expression of constitutively active Rab11 mutant not only led to accumulation of FVIIa and EPCR in the REC, but also resulted in retention of FVIIa and EPCR for a longer period of time within this compartment. It has been shown that hydrolysis of GTP bound to Rab11 GTPase is essential for the recycling of transferrin receptor from the REC back to the cell surface [18], [20]. As the constitutively active mutant does not undergo GTP hydrolysis, the expression of Rab11 constitutively active mutant might lead to the impairment of FVIIa recycling from the REC and therefore, the internalized FVIIa is retained within the REC for a prolonged period. However, it is interesting to note that overexpression of constitutively active Rab11 did not prevent but rather enhanced the transport of FVIIa from the apical to basal side. Consistent with the notion that Rab11 regulates FVIIa transport, overexpression of Rab11 dominant negative mutant markedly reduced FVIIa transport to the basal side.

In contrast to Rab5, Rab4, and Rab11, expression of Rab7, either the constitutively active or dominant negative variant, did not alter the kinetics of EPCR-FVIIa endocytosis or its trafficking. Rab7 acts downstream of Rab5 in regulating the membrane transport from early to late endosomes [31]. The unaltered trafficking of FVIIa and EPCR in cells expressing wild-type, constitutively active or dominant negative Rab7 suggests that Rab7 does not play a significant role in EPCR-mediated FVIIa endocytosis and intracellular trafficking. This is consistent with our earlier observation that most of the endocytosed EPCR-FVIIa complexes are recycled back to the cell surface either from early endosomes or the REC and not directed to lysosomal degradation [3].

Although in the present study, we have limited our investigation to EPCR-mediated FVIIa trafficking, it is likely that Rab GTPases regulate other ligands of EPCR, i.e., FVII, protein C and APC, in a similar fashion. It may be pertinent to note here that our earlier studies showed a similar pattern of internalization and cellular localization of FVII, FVIIa, protein C, and APC [3]. The same study also revealed no notable differences in internalization and cellular localization of FVIIa, APC, and their active-site inhibited counterparts. Therefore, it is unlikely that EPCR-FVIIa-mediated PAR signaling [35] contributes to endocytosis and trafficking of EPCR-FVIIa.

Overall, our data presented herein indicate that Rab GTPase activity plays a role in regulating EPCR and FVIIa levels at the cell surface by controlling the rate at which the receptor and receptor-ligand complex are processed through the endosomal compartments. The ability of Rab GTPases to regulate EPCR trafficking suggests that mutations leading to altered Rab GTPase activity and/or differences in Rab GTPase protein levels may affect EPCR function by altering the dynamics of its endocytosis, intracellular trafficking, and plasma membrane recycling. A number of studies have associated various human diseases with the expression of mutant Rab GTPases [36]–[38]. Further, expression of Rab proteins could vary in different cell types in a variety of pathological conditions [39]–[42]. Therefore, it is possible that differences in Rab protein expression or mutation in Rab GTPases in various cell types and pathological conditions may modulate EPCR expression at the cell surface and intracellular trafficking, which could consequently affect EPCR-mediated anticoagulant and cell signaling functions under various pathological conditions.
